# Supplementary material for: Inhibition of RNA Helicase Activity Prevents Coxsackievirus B3-Induced Myocarditis in Human iPS Cardiomyocytes
Source: Int J Mol Sci. 2020 Apr 25;21(9):3041. doi: 10.3390/ijms21093041 (PMC7246926; doi:10.3390/ijms21093041)
Supplement: Supplementary file 1 [file ijms-21-03041-s001.pdf]

Figure S1. M-mode echocardiograms were performed using CVB3 infected mice.

A

CVB3 infection only

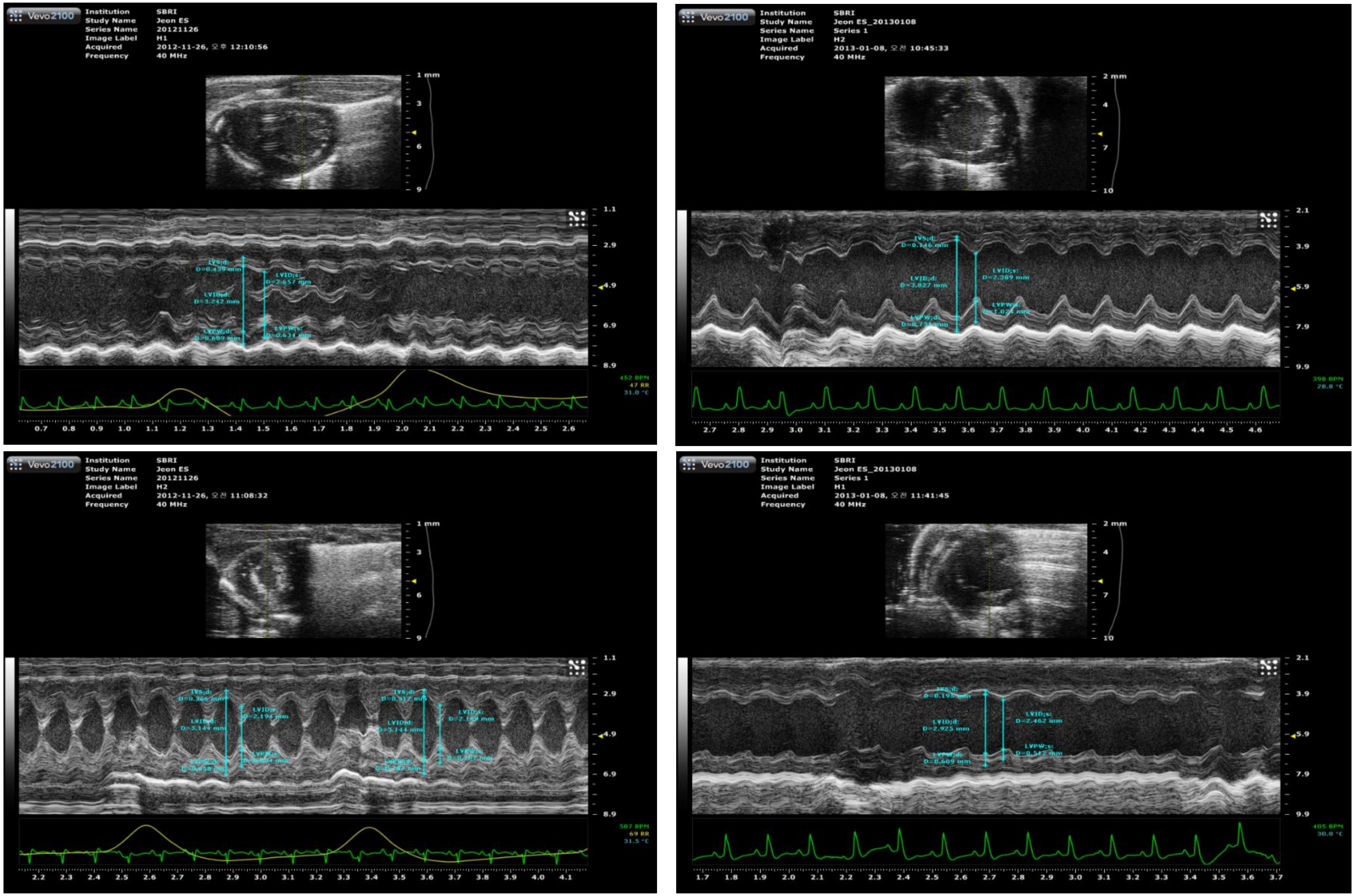

B

CVB3 infection + E2CI

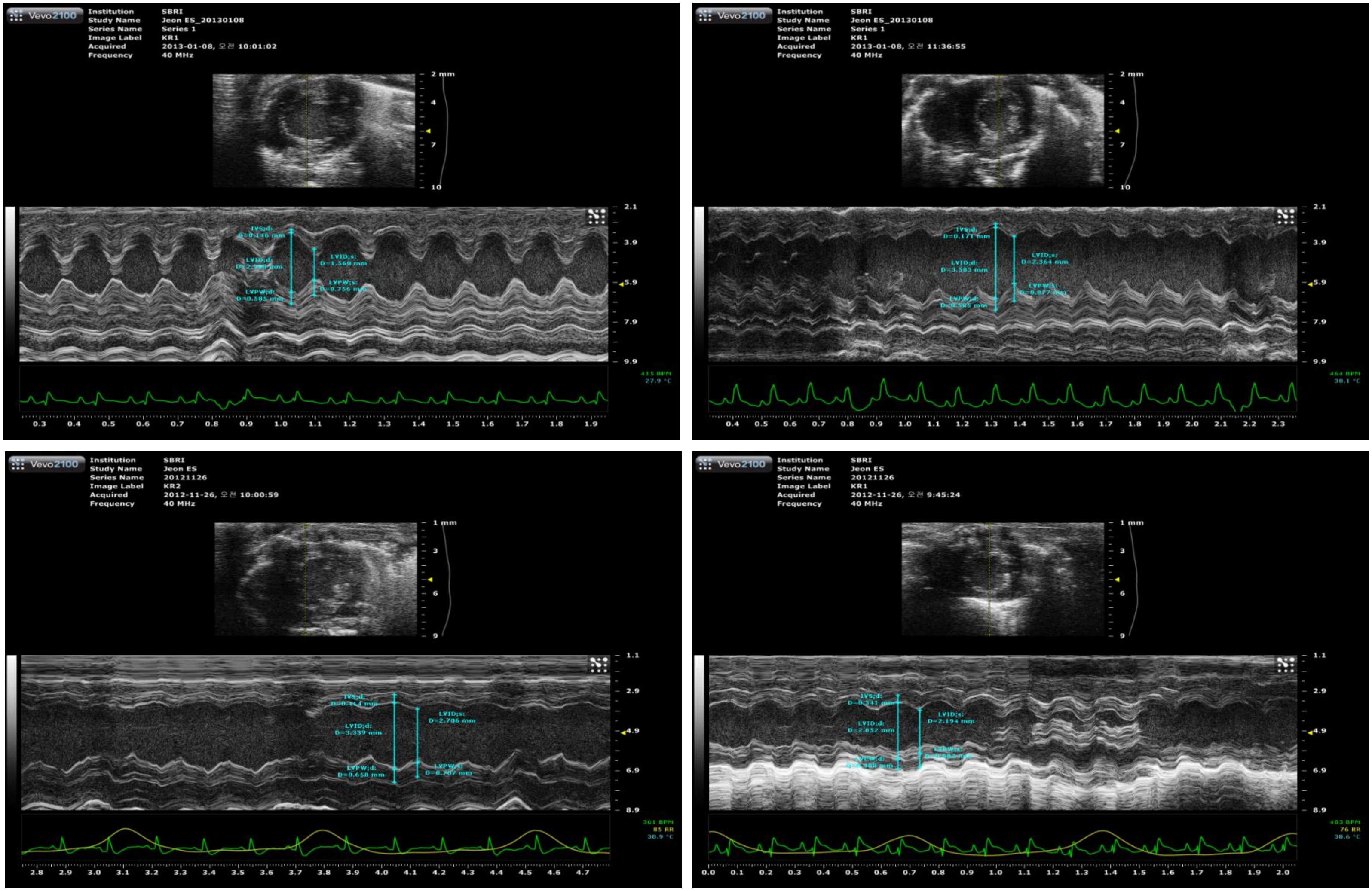

Figure S2. Highly purified human induced pluripotent stem cell-derived cardiomyocytes (iCell Cardiomyocytes®, Cellular Dynamics International, Inc., (CDI), Madison, WI, USA) were used in the experiments. A. Certificate of analysis. B. Identification of iCell cardiomyocytes.

A

Cellular Dynamics international

Cellular Dynamics International, Inc.
525 Science Drive
Madison, WI 53711 USA

Toll Free in US (877) 310-6688 / (608) 310-5100 T
(608) 310-5101 F
support@cellulardynamics.com E
www.cellulardynamics.com W

Certificate of Analysis

**Product Name:**	iCell® Cardiomyocytes	**Lot Number:**	1097923
**Catalog Number:**	CMC-100-010-001	**Viable Cardiomyocytes/Vial:**	5.37×10^6
**Use By:**	12 months from date of shipment		See the quantity information in the Product Terms and Conditions

All iCell Cardiomyocytes are subject to the use restrictions in the iCell Cardiomyocytes User's Guide. Failure to adhere to the instructions within the User's Guide may void your limited warranty.

B

iCell Cardiomyocytes (human IPS induced cardiomyocytes)

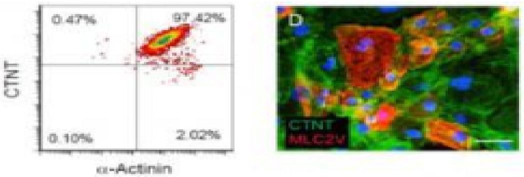

Figure 1: iCell Cardiomyocytes are a High-Purity Cardiac Population.
Flow cytometry analysis and immunostaining show that iCell Cardiomyocytes are typically >95% cTNT+) with intact sarcomeric myofilament organization. (Data were adapted from Kattman et al., 2011.)

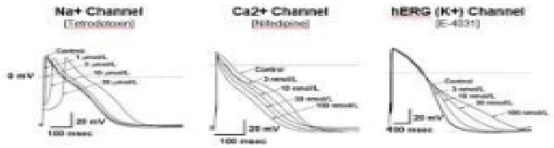

Figure 2: iCell Cardiomyocytes Recapitulate Native Cardiac Function
iCell Cardiomyocytes form a spontaneously beating monolayer within 7 days. iCell Cardiomyocytes contain the expected human cardiac ionic currents and show the expected effects when exposed to compounds including ion channel blockers. (Data were adapted from Ma et al., 2011).

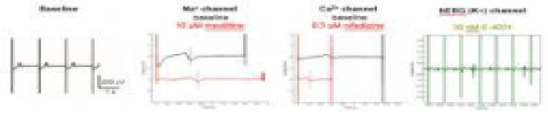

Figure 3: iCell Cardiomyocytes have Appropriate Sarcomeric Organization, Calcium Handling and Intact Excitation-Contraction Coupling.
Their electrophysiological activity can be pharmacologically modulated and quantified by recording the electrical activity using a multielectrode array (MEA). The field potential duration (FPD) increases or decreases as expected when exposed to ion channel-blocking drugs for key cardiac channels.

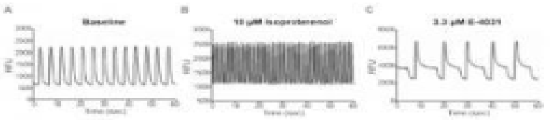

Figure 4: Intracellular Calcium (Ca2+) Handling Provides a High-throughput Biomarker for Ion Channel and GPCR Activity.
Electrical activity at the membrane is controlled by ion channels and GPCRs. This activity drives intracellular Ca2+ handling. Panel A shows representative calcium handling waveforms at baseline. Panels B and C show the effect of the GPCR β-adrenergic agonist ISO or the IKr channel blocker E-4031, respectively.

FUJIFILM Cellular Dynamics, Inc. Madison, WI, USA

References

Schocken D, Stohlman J, Vicente J, Chan D, Patel D, Matta MK, Patel V, Brock M, Millard D, Ross J, Strauss DG, Blinova K. (2018) Comparative Analysis of Media Effects on Human Induced Pluripotent Stem Cell-derived Cardiomyocytes in Proarrhythmia Risk Assessment. J Pharmacol Toxicol Methods (90):39-47.

Bedut S, Seminatore-Nole C, Lamamy V, Caignard S, Boutin JA, Nosjean O, Stephan JP, Coge F. (2016) High-throughput Drug Profiling with Voltage- and Calcium-sensitive Fluorescent Probes in Human iPSC-derived Cardiomyocytes. Am J Physiol Heart Circ Physiol 311(1):H44-53.

Figure S3. Confirmatin of E2CI cytotoxicity. E2CI cytotoxicity was confirmed on human iPS cardiomyocyte at low (10ng/ml- 0.1ng/ml) or high (10ug/ml- 0.1ug/ml) dose.

E2CI antiviral effect without cytotoxicity

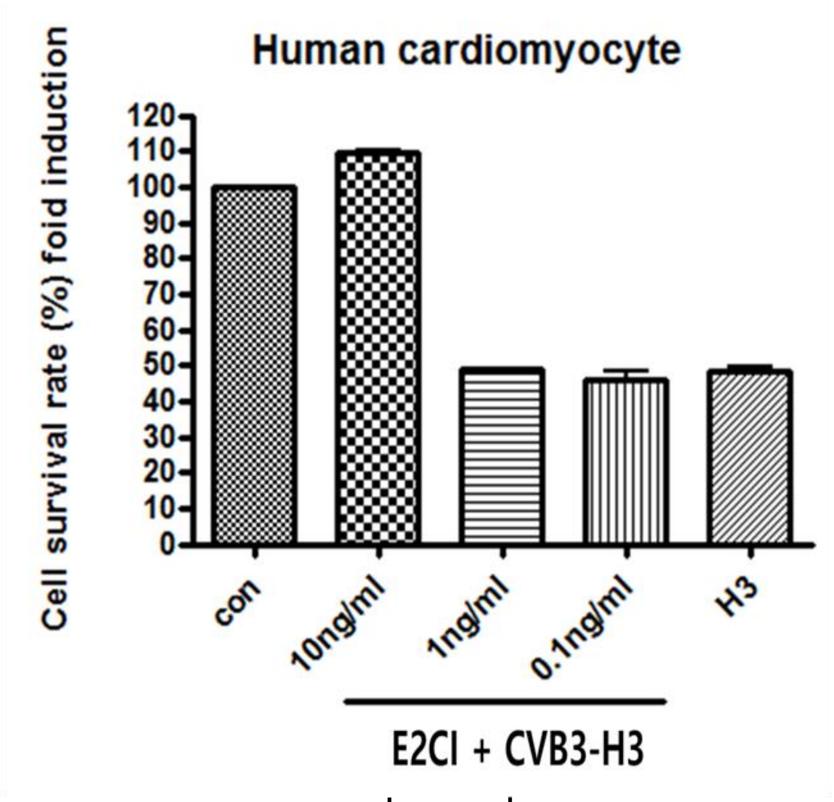

Low dose

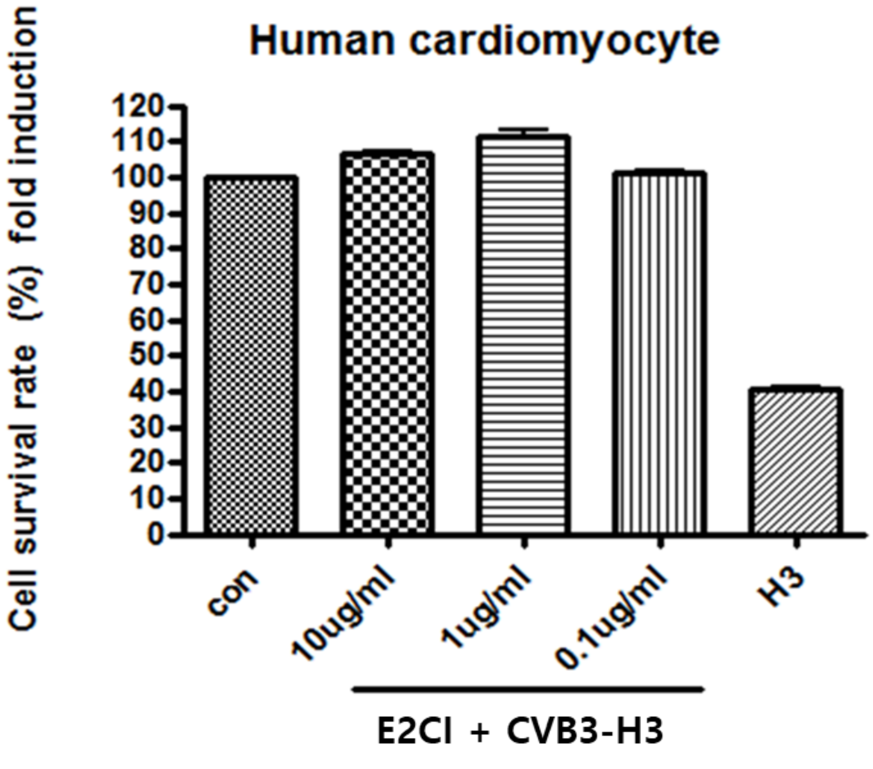

High dose
